# Examining the Relationship Between Perceived Stress and Cognition in Older African American Adults

**DOI:** 10.1002/alz70861_107955

**Published:** 2025-12-23

**Authors:** Isabella Manrique, Robbie Beyl, Robert L Newton, Owen T. Carmichael

**Affiliations:** ^1^ Pennington Biomedical Research Center, Baton Rouge, LA USA

## Abstract

**Background:**

Perceived psychological stress can negatively affect cognition in older adults. However, this relationship is not well studied in Black Americans, even though they experience higher levels of stress due to discrimination and lack of access to resources. The current study investigated the association between perceived stress and cognition in cognitively healthy older Black Americans. We hypothesized that higher perceived stress would be associated with poorer cognition.

**Method:**

112 participants from the Reducing African Americans’ Alzheimer’s Disease Risk Through Exercise (RAATE) study (Mean age = 67.6, 77.6% female) completed the Perceived Stress Scale (PSS) as well as the NIH Toolbox Cognitive Battery and the Rey Auditory Verbal Learning Test (RAVLT). Linear regression models examined the association between PSS score and cognitive test scores, controlling for age along with one additional covariate among gender, education, income, and marital status.

**Result:**

Greater PSS score was associated with lower RAVLT score when controlling for age and gender (b = ‐0.37, *p* = 0.04). Greater PSS score was associated with lower Dimensional Change Card Sort Test score when adjusting for age and education (b = ‐0.44, *p* = 0.04). PSS scores and other NIH Toolbox Cognitive Battery scores were not significantly associated when adjusting for other covariates

**Conclusion:**

Greater perceived stress was associated with poorer memory and executive function when adjusting for demographic factors in a sample of older Black American adults.

